# Effects of Graded Dietary L-Carnitine Levels on Growth Performance, Carcass Traits, and Physiological Responses in Pekin Ducklings

**DOI:** 10.3390/biology15161439

**Published:** 2026-08-20

**Authors:** Assem Safwat, Asmaa Elnaggar, Osama Elghalid, Mahmoud El-Kelawy, Ibrahim Mankola, Birendra Mishra, Karim El-Sabrout

**Affiliations:** 1 Department of Poultry Production, Faculty of Agriculture, Alexandria University, Alexandria 21546, Egypt; 2 Department of Animal and Poultry, Faculty of Agriculture, Damanhour University, Damanhour 22516, Egypt; asmaaelnaggar85@yahoo.com (A.E.);; 3 Department of Poultry Production, Faculty of Agriculture, New Valley University, El-Kharga 72511, Egypt; 4 Department of Human Nutrition, Food and Animal Sciences, University of Hawaii at Manoa, 1955 East-West Road, Honolulu, HI 96822, USA

**Keywords:** antioxidant status, digestibility, ducklings, immune response, L-carnitine, productive performance

## Abstract

This study evaluated the effects of dietary LC inclusion (0, 50, 100, 200, and 400 mg/kg) in Pekin ducklings from 7 to 65 days of age on growth performance, carcass traits, metabolic profile, immune status, and economic efficiency. A total of 250 unsexed 7-day-old Pekin ducklings were randomly allocated to five dietary treatment groups, each comprising five replicates of 10 ducklings. The linear response plateau analysis estimated LC breakpoints of approximately 50 mg/kg diet for body weight gain and feed conversion ratio. Additionally, LC significantly improved carcass yield and crude protein content of the meat, as well as enhanced its antioxidant capacity. Physiologically, LC enhanced hematological indices, lipid metabolism, and thyroid hormone activity, without adversely affecting liver function. Moreover, LC favorably modulated the intestinal microbiota by increasing the abundance of beneficial *Lactobacillus* spp. and reducing pathogenic bacteria. Furthermore, it improved immune status, as indicated by higher immunoglobulin levels, lysozyme and phagocytic activities, and lymphocyte proliferation. LC significantly enhanced the mentioned parameters without inducing any adverse effects; however, the magnitude of the response plateaued and subsequently declined at higher inclusion levels (200–400 mg/kg). The improvements in performance and health status were attributed to the enhanced fatty acid oxidation, energy metabolism, antioxidant defense, and immunomodulatory effects of LC. In conclusion, including 50 mg L-carnitine/kg in the diet represents an effective and appropriate nutritional strategy under the conditions of the present study to optimize growth performance, carcass quality, nutrient digestibility, health status, and economic profitability in Pekin duck production systems.

## Simple Summary

This study mainly aimed to investigate the efficacy of dietary L-carnitine (LC) inclusion—administered at graded levels from 0 to 400 mg/kg between 7 and 65 days of age—in reducing excessive lipid deposition in Pekin duck carcasses. The results demonstrated that moderate dietary LC inclusion improved growth performance, feed conversion ratios, apparent nutrient digestibility, and economic efficiency. Furthermore, LC significantly increased carcass yield, the crude protein content of the meat, and meat antioxidant capacity while reducing abdominal fat percentages. Physiologically, dietary LC inclusion boosted thyroid hormone activity, optimized lipid metabolism, and promoted healthier gut microbiota by increasing beneficial *Lactobacillus* spp. and reducing pathogenic bacteria. It also significantly strengthened both cellular and humoral immune responses—indicated by increased immunoglobulin levels, lysozyme activity, and lymphocyte proliferation—without inducing any adverse effects on liver function. Ultimately, the study concludes that adding LC to duck feed (up to 100 mg/kg) safely and effectively boosts duck growth, meat quality, and overall health.

## 1. Introduction

The rise in global demand for high-quality animal protein is a critical issue, particularly for developing countries that suffer from dietary protein deficiency. Chicken production is vital to meet this growing demand because chickens have an excellent feed conversion efficiency and a faster growth rate than most other farm animal species. In addition, chicken production is relatively cost-effective compared with many other areas of the livestock industry [1]. The duck production industry has attracted considerable attention because of the rapid growth rate, ease of production, and the high market value of duck meat; it constitutes an important segment of the poultry industry in Egypt and many other developing countries [2,3].

However, the adoption of duck meat in the diet may be hindered by its higher fat content relative to broilers, which is identified as one of the main limiting factors. Generally, duck carcasses are characterized by excessive subcutaneous and abdominal fat deposition, which negatively affects carcass quality and product acceptability, particularly among health-conscious consumers seeking leaner meat products [4]. Furthermore, excessive fat deposition in duck meat may contribute to a higher intake of saturated fatty acids, potentially increasing the risk of metabolic syndrome and other cardiometabolic disorders [5]. Therefore, considerable attention has been directed toward developing nutritional strategies that reduce fat deposition while maintaining optimal growth performance and physiological status in poultry. Among these strategies, the use of functional feed additives has shown considerable promise in improving poultry productivity and health [6].

L-carnitine (LC) is a naturally occurring quaternary amine synthesized primarily from the amino acids lysine and methionine. It plays a crucial role in lipid metabolism by facilitating the transport of long-chain fatty acids across the mitochondrial membrane for β-oxidation and subsequent energy production [6,7]. Although poultry can synthesize LC endogenously, its production may be insufficient to meet physiological demands under conditions of rapid growth, environmental or metabolic stress, or limited availability of precursor amino acids [8]. Dietary inclusion of LC has been reported to improve growth performance, feed efficiency, and carcass traits in poultry, primarily through enhancing fatty acid oxidation and reducing fat deposition [9,10]. In this context, LC has been demonstrated to enhance antioxidant defense by increasing the activity of endogenous antioxidant enzymes and mitigating oxidative stress [11]. Furthermore, LC has been reported to modulate immune function by stimulating immunoglobulin production and enhancing immune responses in poultry [5]. It has also been associated with lipid profile improvement by reducing plasma cholesterol and triglyceride concentrations [10,12].

Despite the increasing body of research on LC inclusion in various poultry species, few studies have comprehensively investigated the dose-dependent effects of graded dietary LC inclusion on productive performance, carcass characteristics, metabolic and physiological responses, antioxidant status, immune function, and economic efficiency in Pekin ducklings. Accordingly, this study aimed to determine the optimal dietary LC inclusion level for maximizing production efficiency while improving carcass quality, physiological status, nutrient utilization, and gut microbial balance, thereby providing practical nutritional recommendations for sustainable Pekin duck production.

## 2. Materials and Methods

### 2.1. Experimental Location

The experiment was conducted at the Animal and Poultry Research Center, Faculty of Agriculture, Damanhour University, Egypt.

### 2.2. Chemical Characteristics of L-Carnitine

The L-carnitine used in the present study was purchased from a certified commercial supplier in Damanhour City, Beheira Governorate, Egypt. The product was supplied as feed-grade L-carnitine (free base, ≥98% purity; Merck KGaA, Darmstadt, Germany) in crystalline powder form and was incorporated into the basal diet at the designated inclusion levels after thorough mixing to ensure homogeneity.

### 2.3. Birds and Housing Management

Two hundred and fifty unsexed 7-day-old ducklings of the Pekin breed (*Anas platyrhynchos domesticus*), with an average initial body weight of 163 ± 3.42 g, were used in this study. During the first seven days after hatching, all ducklings were fed a standard commercial starter diet formulated to meet the nutrient requirements of Pekin ducklings, with free access to feed and drinking water. At 7 days of age, birds were individually weighed and randomly allocated to five experimental treatments, each comprising 50 ducklings. Each treatment was further subdivided into five replicate pens of 10 birds each. Five experimental diets were formulated, comprising a basal diet without LC inclusion (control) and four basal diets including LC at 50, 100, 200, and 400 mg/kg diet, respectively. All experimental diets were formulated to meet or exceed the nutrient requirements of growing ducks as recommended by the NRC [13]. Ingredients and calculated nutrient composition of starter and grower diets are illustrated in Table 1.

Feed and water were provided *ad libitum* throughout the experimental period. Birds were reared under standard environmental and managerial conditions. A continuous lighting program of 23 h light per day was applied during the first week, followed by 20 h light per day from the second week until the end of the experiment. Upon arrival, ducklings were brooded at 33 °C, after which the ambient temperature was gradually reduced to 30–27 °C during the second week and further decreased to approximately 24 °C from the third week until 65 days of age, following recommended management guidelines for duck production [14].

### 2.4. Data Collected

#### 2.4.1. Productive Performance

Growth performance traits, including live body weight (LBW), body weight gain (BWG), feed consumption (FC), and feed conversion ratio (FCR), were collected on a replicate-pen basis for each treatment group during the periods of 7–35 d, 36–65 d, and the overall experimental period (7–65 d). European production efficiency index (EPEI) was also computed for production efficiency using the equation provided by Marcu et al. [15] as follows:

$$EPEI=\left(\frac{Livability\;\left(\%\right)\times \;Final\;body\;weight\left(kg\right)}{Age\left(d\right)\times FCR\;(kg\;feed/kg\;gain)}\right)\times 100$$

where:

FCR = feed conversion ratio.

Economic evaluation was conducted based on prevailing local market prices of feed ingredients and live body weight at the time of the experiment. Economic efficiency (EE) was calculated as the ratio of net revenue to total production cost and expressed as a percentage. Relative economic efficiency (REE) was determined by comparing the economic efficiency of each treatment group to that of the control group, following standard procedures used in poultry production economics [16].

#### 2.4.2. Digestibility Trial

At the end of the experimental period (65 d of age), a digestibility trial was carried out to estimate the apparent nutrient digestibility coefficients of the experimental diets. Five healthy representative ducklings per treatment were randomly selected for the digestibility trial and individually housed in metabolic cages (42 × 50 × 50 cm) equipped with separate feeders and drinkers. The number of birds used for the digestibility trial was determined based on the housing capacity of the experimental facility and the fixed experimental design established before the study. Birds were maintained under the same environmental conditions as the growth trial, with *ad libitum* access to feed and fresh drinking water. The house temperature was maintained at 24 ± 2 °C, and a 20 h light: 4 h dark lighting program was applied throughout the digestibility trial. The digestibility trial lasted 11 days, comprising a 7-day adaptation period followed by a 4-day total excreta collection period, in accordance with standard procedures commonly used in poultry digestibility studies, following the classical formula [17]:

$$Apparent\;nutrient\;digestibility\;(\%)=\left(\frac{Nutrient\;intake-Nutrient\;excreted}{Nutrient\;intake}\right)\times 100$$

During the collection period, feed consumption and total excreta output were quantitatively recorded and weighed individually for each bird on a daily basis. Total excreta collected from each bird were pooled over the collection period, thoroughly homogenized, oven-dried to a constant weight, ground to pass through a 1-mm sieve, and stored in sealed containers until subsequent analysis. Chemical analyses of feed and excreta samples were conducted for crude protein (CP), crude fiber (CF), ether extract (EE), and ash content using the official methods of AOAC [18]. Apparent CP digestibility was corrected for uric acid nitrogen excretion to provide a more accurate estimate of nitrogen utilization [17]. Nitrogen-free extract (NFE) was calculated on a dry matter (DM) basis by subtracting CP, CF, EE, and ash content from 100.

#### 2.4.3. Carcass Characteristics and Chemical Composition of Meat

At the end of the experiment (65 days of age), five ducks from each treatment group (one bird per replicate) were randomly selected. Selected birds were overnight-fasted, individually weighed, and humanely slaughtered by severance of the jugular vein in accordance with Islamic slaughter procedures and approved ethical guidelines. Following evisceration, the carcass was opened, and the eviscerated carcass, along with internal organs, including the liver, gizzard, pancreas, abdominal fat, spleen, thymus, and bursa of Fabricius, was individually weighed. Relative organ and carcass weights were calculated as a percentage of the pre-slaughter live body weight. Carcass percentages were calculated as follows:

Carcass % = (carcass weight/pre-slaughter live body weight) × 100

Dressing % = [(carcass weight + edible parts weight)/pre-slaughter live body weight] × 100

Edible parts weight, g = liver + gizzard + heart

Breast (*Pectoralis major*) and thigh muscle samples were collected immediately after carcass dissection, individually labeled, and stored at −20 °C until analysis. The chemical composition of both muscles was determined by measuring CP and EE according to the AOAC Official Methods of Analysis [18]. The total antioxidant capacity (TAC) of breast and thigh muscle homogenates was determined using the oxygen radical absorbance capacity (ORAC) assay [19] according to the manufacturer’s instructions for the commercial assay kit (Cell Biolabs, Inc., San Diego, CA, USA). Fluorescence intensity was measured using a Synergy HTX Multi-Mode Microplate Reader (BioTek Instruments, Winooski, VT, USA).

#### 2.4.4. Microbiological Analysis of Intestinal Contents

At 65 days of age, intestinal contents were collected from the ileum of 25 ducks (five per treatment) immediately after slaughter for microbiological analysis. Approximately 1 g of ileal digesta was aseptically transferred into sterile tubes containing 9 mL sterile physiological saline (0.85% NaCl) and homogenized to obtain a 10^−1^ dilution. Serial ten-fold dilutions were subsequently prepared for microbiological enumeration using the spread-plate technique on selective media. Microbiological analyses were initiated immediately after sample collection. Total bacterial count (TBC) was determined by plating intestinal content samples on Plate Count Agar (PCA), followed by aerobic incubation at 37 °C for 48 h. *Lactobacillus* spp. were enumerated on de Man, Rogosa and Sharpe (MRS) agar after anaerobic incubation at 37 °C for 48 h. *Escherichia coli* (*E. coli*) was quantified on MacConkey agar, whereas *Proteus* spp. were enumerated on Xylose Lysine Deoxycholate (XLD) agar following aerobic incubation at 37 °C for 24 h. After incubation, bacterial colonies were identified based on their characteristic morphological features on the respective selective media and enumerated according to the procedures described by Baurhoo et al. [20] and Yadav and Jha [21]. The microbiological assessment was specifically designed to quantify cultivable and viable bacterial populations; therefore, PCR-based molecular identification and additional biochemical characterization were not performed. Microbial populations were expressed as colony-forming units (CFU)/g of intestinal contents.

#### 2.4.5. Blood Collection and Hematobiochemical Analyses

At the termination of the experiment (65 days of age), blood samples (approximately 3 mL) were withdrawn from the brachial vein of five birds per treatment that were different from those selected for carcass evaluation. Each sample was collected into two parts; the first part was retained without anticoagulant to obtain serum, and the second part was kept in a heparinized tube to estimate the complete blood count. Serum was separated by centrifugation at 4000 rpm for 15 min and stored at −20 °C until subsequent biochemical analysis.

Red blood cell (RBC) and white blood cell (WBC) counts, together with the differential leukocyte count, were determined using standard hematological procedures [22]. Concentration of hemoglobin (Hb) and packed cell volume percentage (PCV) were estimated as described by Provan et al. [23]. Determination of the concentration of glucose, total protein, albumin, total lipid, triglyceride (TG), cholesterol, high-density lipoprotein (HDL), low-density lipoprotein (LDL), aspartate aminotransferase (AST), alanine aminotransferase (ALT), uric acid, creatinine, TAC, glutathione peroxidase (GSH-Px), superoxide dismutase (SOD), malondialdehyde (MDA), triiodothyronine (T_3_), thyroxine (T_4_), and IgG, IgM and IgA fractions was made by special kits: sentinel CH (Sentinel Diagnostics, Milan, Italy), CAL-TECH Diagnostics, Inc. (Chino, CA, USA) using a Beckman DU-530 spectrophotometer (Hanau, Germany), Diagnostic Products Corporation (Los Angeles, CA, USA), and Reactivos GPL (Barcelona, Spain), following manufacturer’s recommendations.

Phagocytic index and activity (PI and PA) were determined using the method of Leigh et al. [24]. Serum bactericidal activity (BA) on the strain of *Aeromonas hydrophila* was determined according to Rainger and Rowley [25]. Serum lysozyme activity (LA) was evaluated by the turbidimetric method as described by Engstad et al. [26], wherein one unit of LA is defined as an absorbance decreasing at 0.001/min. The lymphocyte transformation test (LTT) was carried out following the method of Balhaa et al. [27].

### 2.5. Statistical Analysis

Data were analyzed as a completely randomized design using the General Linear Model (GLM) procedure of the SAS program 9.4 (SAS Institute Inc., Cary, NC, USA). Percentage data were transformed using either the square-root or arcsine transformation, as appropriate, whereas bacterial counts were log_10_-transformed before analysis to satisfy the assumptions of normality and homogeneity of variance. Prior to statistical analysis, model residuals were tested for normality using the Shapiro–Wilk test, and homogeneity of variance among treatments was assessed using Levene’s test. Additionally, orthogonal polynomial contrasts were performed to evaluate the linear and quadratic responses of the measured variables to increasing dietary LC levels. When a significant treatment effect was detected, differences among treatment means were evaluated using Duncan’s multiple range test [28]. To determine the optimum dietary LC requirement, data for BWG and FCR were fitted to a one-slope linear response plateau (LRP) regression model defined as $Y=L+U\times \left(R-X\right)$, where L represents the plateau value, U is the response slope, R is the estimated requirement breakpoint, and X is the dietary LC concentration. The breakpoint R was defined as the threshold beyond which additional LC supplementation yielded no further biological performance gains. Statistical significance was declared at *p* ≤ 0.05. The following statistical model was applied:

Y_ij_ = µ + T_i_ + e_ij_

where Y_ij_ = an observation, μ = overall mean, T_i_ = effect of treatment (i = 1, 2, …, 5), and e_ij_ = experimental random error.

## 3. Results

### 3.1. Productive Performance Traits

Dietary inclusion of LC significantly improved the growth performance of Pekin ducklings throughout the experimental period (*p* < 0.05; Table 2). Initial body weight did not differ among the experimental groups (*p* = 0.989), confirming comparable baseline conditions. Compared with the control group, dietary LC increased final body weight and BWG by approximately 12.3–19.1% and 12.8–20.0%, respectively. In addition, LC inclusion reduced FC by 4.2–8.4% and improved FCR by 9.4–23.7% relative to the control.

Economic parameters closely reflected productive performance. EE was significantly increased (*p* = 0.002), peaking at 50 and 100 mg LC inclusion (95.7% and 94.3%, respectively) compared with the control group (62.5%), representing increases of 53.0% and 50.9%, respectively. Similarly, the EPEI was significantly improved (*p* = 0.0001), with the highest value recorded for the 50 mg LC inclusion treatment, followed by the 100 mg LC group, with 69.8% and 60.6%, respectively, higher than the control.

**Figure 1:**
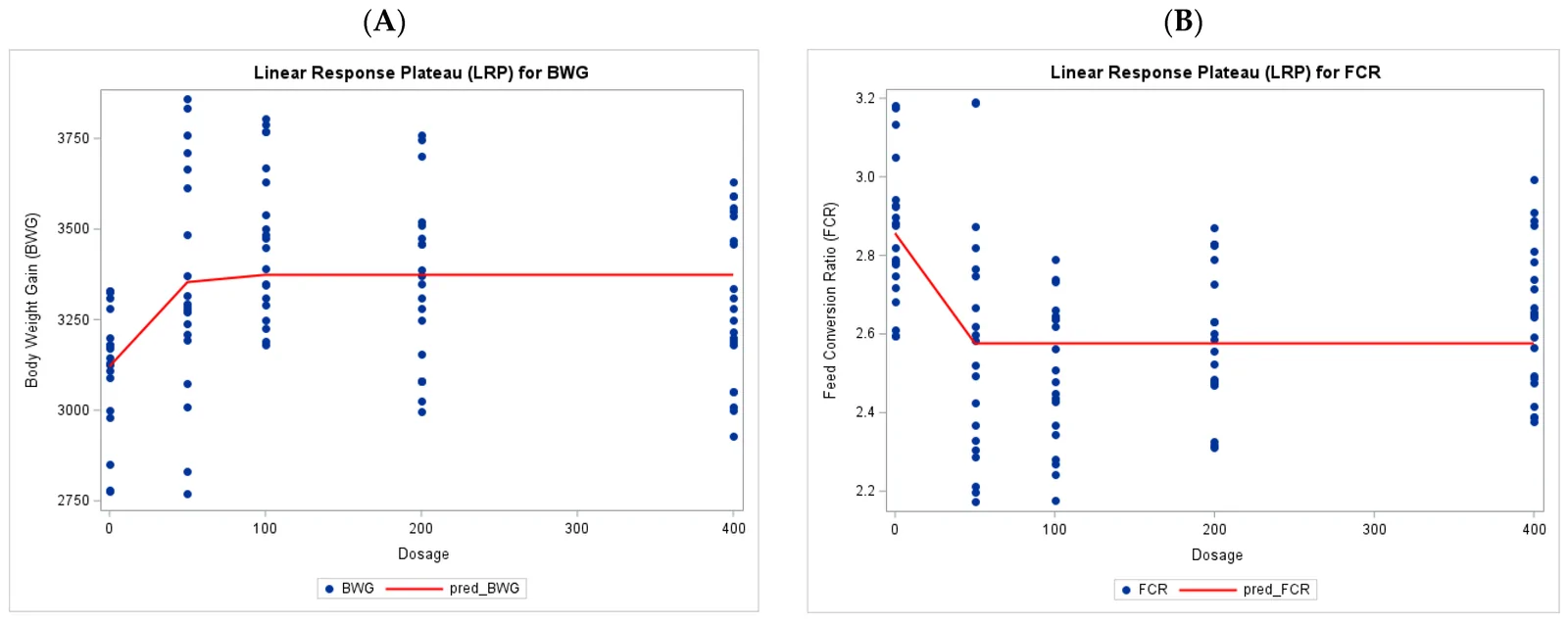
Fitted linear response plateau (LRP) regression models of body-weight gain ((**A**); BWG (g)) and feed conversion ratio ((**B**); FCR (g:g)) as a function of dietary L-carnitine (LC) level. The estimated LC requirement determined by the one-slope LRP analysis was 54.33 mg/kg diet for BWG [Y = 3373.6 − 4.65 (54.33 − X)] and 50.00 mg/kg diet for FCR [Y = 2.5758 + 0.00560 (50.00 − X)].

The dose–response relationships between the different LC levels and BWG and FCR determined by LRP regression are illustrated in Figure 1A,B. Non-linear regression analysis using an LRP model revealed requirement breakpoints at 54.33 mg/kg for BWG and 50.00 mg/kg for FCR.

### 3.2. Apparent Nutrient Digestibility

At 65 days of age, ducklings fed diets containing LC from 7 days of age exhibited significantly higher apparent digestibility of most nutrients than the control group (*p* < 0.05; Table 3). Compared with the control group, OM, CP, EE, and NFE digestibility increased by approximately 8.4–11.1%, 7.7–13.8%, 10.9–14.2%, and 6.0–10.6%, respectively. However, CF digestibility was not influenced by dietary LC (*p* > 0.05). Although dietary LC inclusion at all tested levels generally improved the measured parameters compared with the control, the response at 400 mg LC/kg diet was often less pronounced than that observed at the lower inclusion levels (50–200 mg/kg).

### 3.3. Carcass Traits and Meat Composition

Table 4 demonstrates that dietary inclusion of LC significantly improved carcass characteristics and meat quality traits in Pekin ducklings (*p* < 0.05). Compared with the control group, LC increased carcass yield and dressing percentage by approximately 6.8–9.9% and 4.2–7.5%, respectively, while reducing abdominal fat deposition by 52.7–55.0%. In contrast, no significant effects were observed on the relative weights of the visceral or lymphoid organs (*p* > 0.05).

Dietary LC inclusion significantly improved the chemical composition of duck meat by increasing crude protein content (*p* = 0.001) and reducing intramuscular fat content (*p* = 0.002), relative to the control group. Furthermore, TAC of duck meat was significantly increased by LC inclusion (*p* = 0.001).

### 3.4. Microbiological Intestinal Contents

As shown in Table 5, dietary LC inclusion significantly modulated the intestinal microbial community of Pekin ducklings (*p* < 0.05). Compared with the control group, total bacterial count, *Escherichia coli*, and *Proteus* spp. populations were reduced in LC-treated birds by approximately 7.7–16.1%, 17.1–29.1%, and 29.8–43.1%, respectively, whereas *Lactobacillus* spp. counts increased by 34.0–41.8%.

### 3.5. Hematological Parameters and Biochemical Constituents

Dietary LC inclusion significantly (*p* < 0.05) improved several hematological parameters in Pekin ducklings (Table 6). Compared with the control group, RBC count, Hb concentration, PCV, WBC count, and lymphocyte percentage increased by approximately 23–29%, 42–53%, 12–20%, 18–23%, and 10–15%, respectively. In contrast, LC inclusion did not significantly affect the proportions of other leukocyte types, including heterophils, or the heterophil-to-lymphocyte (H/L) ratio (*p* > 0.05).

Table 7 shows that all LC-treated groups exhibit significantly greater total protein and globulin concentrations (*p* = 0.001) than the control group, whereas albumin concentration was not significantly affected (*p* = 0.081). The greatest numerical increases in total protein (24.04%) and globulin (23.50%) were observed in ducks treated with 100 mg LC relative to the control.

Dietary LC inclusion significantly altered the serum lipid profile of Pekin ducklings (*p* < 0.05). Compared with the control group, serum total lipid, cholesterol, and LDL concentrations were significantly reduced in all LC-treated groups by approximately 10–18%, 8–10%, and 16–19%, respectively, while HDL concentration was significantly increased in all treated groups (*p* = 0.002) by around 13–16%. On the other hand, triglyceride levels were significantly increased in all treated groups (*p* = 0.002) by approximately 8–12%.

There was no significant effect of dietary LC inclusion on serum hepatic enzyme activities, including AST, ALT, and ALP (*p* > 0.05). In contrast, serum creatinine and uric acid concentrations decreased by approximately 20–30% and 14–24%, respectively. Dietary LC also increased serum glucose and thyroid hormones (T_3_ and T_4_).

### 3.6. Antioxidative Status and Immunological Indices

As presented in Table 8, dietary LC inclusion significantly enhanced the antioxidant status of Pekin ducklings (*p* < 0.05). TAC, GSH-Px, and SOD activities were significantly increased by approximately 9–12%, 7–10%, and 10–13%, respectively, whereas MDA concentration was reduced (*p* = 0.001) by approximately 6–12% compared with the control group, indicating reduced lipid peroxidation and enhanced antioxidant defense.

As shown in Table 8, dietary LC enhanced both humoral and cellular immune responses. Compared with the control group, serum IgG, IgM, and IgA concentrations increased by approximately 3–13, 8–12, and 19–23%, respectively. Similarly, PA, BA, LA, and the LTT responses were significantly improved by 13–16, 9–15, 16–25, and 17–29%, respectively, in LC-treated ducks. Conversely, the PI decreased in all LC-treated groups compared to the control by 16–18%.

## 4. Discussion

The results demonstrate that dietary LC inclusion at 50 to 100 mg/kg diet effectively improved productive performance in Pekin ducklings, particularly LBW, BWG, FCR, and EE. As illustrated in Figure 1, the breakpoints confirm that the biological maximum is achieved at moderate inclusion levels (50–100 mg/kg), after which performance enters a plateau. This further confirms that dietary supplementation at 50 mg/kg is sufficient to optimize growth and feed efficiency without incurring unnecessary feed additive costs. However, increasing the dietary LC level beyond this range (200–400 mg/kg diet) produced only marginal additional benefits, with most productive responses reaching a plateau or showing a slight decline, suggesting that moderate LC inclusion is sufficient to maximize performance. These findings agree with those of Tufarelli et al. [29], who demonstrated that increasing dietary LC beyond the optimal level resulted in diminishing marginal benefits, with productive responses eventually reaching a plateau.

The improvement in LBW and BWG observed in the present study may be attributed to the fundamental role of dietary LC in metabolism process. LC facilitates the transport of long-chain fatty acids into the mitochondrial matrix for β-oxidation, thereby enhancing energy production and improving nutrient utilization efficiency [30,31]. The increased availability of metabolic energy supports protein synthesis and muscle accretion, ultimately promoting growth and productive performance. Furthermore, LC inclusion has been reported to stimulate circulating insulin-like growth factor-1 (IGF-1) concentrations, which may further contribute to enhanced growth and tissue development [5,32].

The improvement observed in the present study in nutrient digestibility, dressing percentage, and total edible parts, while reducing abdominal fat deposition, may also be attributed to the beneficial effects of dietary LC on digestive efficiency and nutrient utilization. LC plays a pivotal role in optimizing cellular energy metabolism while sparing essential amino acids, particularly lysine and methionine, thereby enhancing nitrogen utilization for protein synthesis and other anabolic processes [33,34]. Furthermore, LC has been reported to stimulate digestive enzyme activity, enhance intestinal nutrient absorption, and favorably modulate the gastrointestinal microbiota, thereby promoting microbial fermentation and overall nutrient utilization [35,36]. Similar beneficial effects of LC inclusion on carcass yield and abdominal fat reduction have been reported in ducks, broiler chickens, and quails by Arslan [37], El-Kelawy and Elnaggar [38], and Sarica et al. [39]. LC has been reported to stimulate the activity of carnitine palmitoyltransferase, thereby accelerating fatty acid flux through oxidative pathways and further reducing body fat accumulation [33,40].

Additionally, dietary LC inclusion improved meat composition by increasing CP content and TAC while reducing fat content. These improvements may be attributed to enhanced amino acid utilization for protein synthesis, together with the inhibitory effects of LC on lipogenic enzymes, including glucose-6-phosphate dehydrogenase and lipoprotein lipase, thereby reducing lipid deposition in muscle tissue [29,41,42]. The enhancement in meat quality observed in the present study is consistent with the findings of Awad et al. [43] and El-Kelawy and Elnaggar [38], who also reported improvements in meat quality and protein content following dietary LC inclusion. In contrast, Parsaeimehr et al. [44] and Hassan et al. [5] found no significant effects of LC on meat composition in broiler chickens. These inconsistencies among studies may be attributed to differences in LC inclusion level, poultry species or breed, dietary composition, experimental conditions, and management practices.

Dietary inclusion of LC, particularly at levels of 50–100 mg/kg diet, positively influenced the hematological profile of Pekin ducklings, reflecting improved oxygen-carrying capacity, physiological status, and immune competence. Moreover, the antioxidant properties of LC may protect blood cells from oxidative damage, contributing to improved hematological status [45]. The observed increases in WBC and lymphocyte counts further suggest that LC enhances immune function by promoting leukocyte proliferation and supporting cell-mediated immune responses [8]. The present findings are consistent with previous studies demonstrating the beneficial effects of dietary LC on hematological parameters in poultry. In this context, El-Kelawy and Elnaggar [38], Ikbal and Tekeli [46], and Liu et al. [47] demonstrated that dietary LC inclusion significantly increased RBC count, Hb concentration, PCV, total WBC count, and lymphocyte percentage in broiler chickens. Similarly, Behnam et al. [48] observed significant improvements in Hb concentration and lymphocyte counts following LC inclusion in ducks.

In the present study, LC inclusion improved the serum lipid profile by reducing total lipids, total cholesterol, and LDL concentrations, consistent with previous reports in poultry [5,38,49]. These hypolipidemic effects are likely attributable to the fundamental role of LC in enhancing lipid catabolism, reducing lipid accumulation, and promoting the conversion of cholesterol into bile acids, which increases biliary cholesterol excretion [50]. Similar reductions in serum cholesterol concentrations following dietary LC inclusion have also been reported by Awad et al. [43]. Moreover, El-Kelawy and Elnaggar [38] observed that broiler chickens treated with 50 mg LC/kg diet exhibited the most favorable serum lipid profile, supporting the beneficial effects of moderate LC inclusion on lipid metabolism. Notably, serum triglyceride concentration increased in the LC-treated groups. This response may reflect enhanced lipid turnover rather than impaired lipid metabolism. The role of LC in fatty acid mobilization and mitochondrial β-oxidation can increase hepatic secretion of triglyceride-rich lipoproteins for transport to peripheral tissues during periods of rapid growth [8]. Furthermore, the improved EE digestibility observed in the present study may have increased intestinal lipid absorption, contributing to the modest elevation in circulating triglycerides [49].

Moreover, dietary LC inclusion significantly increased serum glucose, T_3_, and T_4_ concentrations, indicating enhanced metabolic activity in Pekin ducklings. These findings are in agreement with El-Kelawy and Elnaggar [38], who also reported significant increases in serum glucose and thyroid hormone concentrations in LC-treated broiler chickens at 50 mg LC/kg diet, whereas the present study demonstrated that T_3_ continued to increase with higher LC inclusion levels (up to 400 mg LC/kg diet). Similarly, Ahmadipour et al. [45] observed elevated serum glucose concentrations with LC inclusion in growing chicks. The increased glucose and thyroid hormone concentrations may reflect the role of LC in supporting the higher metabolic demands associated with growth. Additionally, the antioxidant properties of LC may help preserve thyroid function by reducing oxidative stress and maintaining cellular integrity. These beneficial effects are likely attributable to the ability of LC to indirectly enhance scavenging of reactive oxygen species and preserve endogenous antioxidant defense systems, thereby protecting tissues from oxidative damage [8]. These findings are consistent with those of Fujimoto et al. [51] and Behnam et al. [48], who reported that LC inclusion increased GSH-Px, SOD, catalase, and TAC, while reducing oxidative stress in poultry species.

Regarding liver and kidney functions, LC inclusion exerted beneficial effects on hepatic and renal function. In the present study, LC reduced serum uric acid and creatinine concentrations without adversely affecting hepatic enzyme activities, indicating improved renal function while maintaining normal liver function. These findings are consistent with previous studies by Ghoreyshi et al. [9], Ismail and Ouda [42], and Tufarelli et al. [29], which reported favorable effects of LC on hepatic and renal biomarkers in poultry.

LC inclusion enhanced both humoral and cell-mediated immune responses in ducklings, as evidenced by increased immunoglobulin concentrations, PA, BA, LA, and LTT. These findings are consistent with previous studies demonstrating that LC improves immune function in poultry through the enhancement of both innate and adaptive immune responses [8,52]. The immunomodulatory effects of LC are likely attributable to its vital role in helping provide adequate energy for immune cell proliferation and activation, together with its antioxidant properties that reduce oxidative stress and preserve the functional integrity of immune cells [6,8]. Similar improvements in humoral and cellular immune responses as affected by dietary LC inclusion have been reported in broiler chickens by El-Kelawy and Elnaggar [38] and Liu et al. [47]. Additionally, Abouzed et al. [53] demonstrated that dietary LC inclusion at 75 and 100 mg/kg diet significantly increased serum IgG concentrations. Similarly, Hassan et al. [5] reported that LC inclusion increased the relative weights of lymphoid organs (spleen and thymus) and enhanced antibody titers against Newcastle disease and avian influenza in broiler chickens. Consistent with the present findings, El-Kelawy and Elnaggar [38] reported that dietary LC inclusion significantly increased serum immunoglobulin concentrations (IgG, IgM, and IgA), and globulin fractions (alpha-, beta-, and gamma-globulins), with the greatest responses observed at 50 mg LC/kg diet.

Despite the positive outcomes observed, certain limitations must be noted. First, microbiological analysis was restricted to standard culture methods without the use of polymerase chain reaction (PCR) or detailed biochemical assays to identify specific microbial species and metabolic pathways. Second, the sample size for some studied traits may have limited the statistical power required to detect subtle treatment differences across some evaluated parameters. Therefore, future studies incorporating advanced molecular pathways and broader biological evaluations on larger populations will be crucial to clarify the precise mechanisms through which LC optimizes ducklings’ health and performance.

## 5. Conclusions

Dietary LC inclusion positively influenced growth performance, nutrient digestibility, carcass quality, antioxidant status, immune response, and economic efficiency in Pekin ducklings. The most consistent and pronounced benefits were achieved at 50 mg LC/kg diet, particularly for productive traits, such as FCR. These findings support the use of LC as a safe and effective functional feed additive for optimizing duck health and farm productivity. However, future studies are needed to evaluate other LC inclusion levels across different duck species and environmental conditions.

## Figures and Tables

**Table 1:** Ingredient composition and calculated nutrient content of the control basal diets.

Ingredients (%)	Starter Period (7–35 Days)	Grower Period (36–65 Days)
Yellow corn	56.80	68.70
Soybean meal (44%)	38.00	26.00
Limestone	1.00	1.05
Dicalcium phosphate	1.90	1.95
Salt (NaCl)	0.30	0.30
Vit + Min.premix ^1^	0.30	0.30
DL-Methionine	0.11	0.15
Sunflower oil	1.50	1.50
Antifungal	0.09	0.05
Total	100.0	100.0
**Nutrient composition (% on DM basis)**		
ME, kcal/kg ^2^	2884	2999
Crude protein ^3^	21.6	17.34
Crude fiber ^3^	3.92	3.37
Ether extract ^3^	3.95	4.30
Lysine ^2^	1.18	0.90
Methionine ^2^	0.44	0.39
Meth. + Cyst. ^2^	0.79	0.69
Calcium ^2^	0.92	1.00
Total phosphorus ^2^	0.78	0.47
Available phosphorus ^2^	0.52	0.31

^1^ The vitamin–mineral premix supplied per kg of diet: vitamin A, 12,000,000 IU; vitamin D_3_, 5,000,000 IU; vitamin E, 80,000 mg; vitamin K_3_, 4000 mg; vitamin B_1_, 4000 mg; vitamin B_2_, 9000 mg; vitamin B_6_, 4000 mg; vitamin B_12_, 20 mg; pantothenic acid, 15,000 mg; nicotinic acid, 60,000 mg; folic acid, 2000 mg; biotin, 150 mg; choline chloride, 400,000 mg; Cu (as copper sulfate), 15,000 mg; I (as calcium iodide), 1000 mg; Fe (as ferrous sulfate), 40,000 mg; Mn (as manganese oxide), 100,000 mg; Zn (as zinc oxide), 100,000 mg; Se (as sodium selenite), 300 mg. ^2^ Calculated. ^3^ Analyzed.

**Table 2:** Influence of different dietary inclusion levels of L-carnitine on growth performance and economic parameters of ducklings during 7–65 days of age (n = 5 birds/treatment).

Item	Control	L-Carnitine (mg/kg)				SEM	*p*-Value		
		50	100	200	400		Treatment	Linear	Quadratic
Initial body weight (g)	162	163	162	162	163	1.33	0.989	0.145	0.119
Final body weight (g)	3240 ^b^	3860 ^a^	3820 ^a^	3700 ^a^	3640 ^a^	8.99	0.001	0.003	0.120
Body weight gain (g)	3080 ^b^	3697 ^a^	3655 ^a^	3538 ^a^	3475 ^a^	7.78	0.002	0.001	0.098
Feed consumption (g)	9500 ^a^	8700 ^c^	8800 ^c^	9000 ^b^	9100 ^b^	8.19	0.001	0.002	0.001
Feed conversion ratio (g feed/g gain)	3.08 ^a^	2.35 ^c^	2.41 ^c^	2.54 ^c^	2.79 ^b^	0.187	0.001	0.0001	0.002
EE, %	62.54 ^c^	95.67 ^a^	94.34 ^a^	88.56 ^b^	87.98 ^b^	2.87	0.002	0.001	0.003
REE, %	100	152.97	150.85	141.61	140.68	--	--	--	--
EPEI, %	148.65 ^c^	252.35 ^a^	238.67 ^a^	226.54 ^ab^	213.85 ^b^	8.44	0.0001	0.0001	0.094

^a,b,c^, Means in the same row followed by different letters are significantly different at *p* ≤ 0.05. SEM, standard error of mean; EE, economic efficiency; REE, relative economic efficiency; EPEI, European production efficiency index.

**Table 3:** Influence of different dietary inclusion levels of L-carnitine on apparent nutrient digestibility (%) of ducklings during 7–65 days of age (n = 5 birds/treatment).

Item	Control	L-Carnitine (mg/kg)				SEM	*p*-Value		
		50	100	200	400		Treatment	Linear	Quadratic
OM	66.7 ^b^	73.2 ^a^	74.1 ^a^	72.9 ^a^	72.3 ^a^	2.38	0.001	0.001	0.065
CP	65.82 ^b^	73.67 ^a^	74.87 ^a^	73.01 ^a^	70.87 ^a^	2.19	0.001	0.002	0.0984
EE	63.98 ^b^	72.98 ^a^	73.09 ^a^	71.97 ^a^	70.98 ^a^	2.23	0.002	0.001	0.108
CF	36.09	37.11	35.98	37.02	36.98	1.38	0.098	0.0891	0.0811
NFE	65.98 ^b^	71.69 ^a^	72.98 ^a^	70.12 ^a^	69.94 ^ab^	2.35	0.001	0.002	0.0941

^a,b^, Means in the same row followed by different letters are significantly different at *p* ≤ 0.05. SEM, standard error of mean; OM, organic matter; CP, crude protein; EE, ether extract; CF, crude fiber; NFE, nitrogen-free extract.

**Table 4:** Influence of different dietary inclusion levels of L-carnitine on carcass characteristics, lymphoid organs, and meat composition of ducklings during 7–65 days of age (n = 5 birds/treatment).

Item	Control	L-Carnitine (mg/kg)				SEM	*p*-Value		
		50	100	200	400		Treatment	Linear	Quadratic
**Carcass traits and organ weights**									
Carcass (%)	65.44 ^b^	69.87 ^a^	71.91 ^a^	69.87 ^a^	70.87 ^a^	2.87	0.002	0.002	0.091
Dressing (%)	68.9 ^b^	71.78 ^a^	73.5 ^a^	73.9 ^a^	74.1 ^a^	1.11	0.001	0.001	0.071
Liver (%)	1.87	1.90	1.78	1.83	1.86	0.087	0.098	0.0911	0.112
Gizzard (%)	2.55	2.45	2.50	2.43	2.41	0.084	0.088	0.098	0.091
Pancreas (%)	0.322	0.312	0.322	0.342	0.326	0.006	0.087	0.0901	0.109
Abdominal Fat (%)	2.98 ^a^	1.34 ^b^	1.37 ^b^	1.41 ^b^	1.41 ^b^	0.087	0.001	0.0002	0.0987
**Lymphoid organs**									
Spleen (%)	0.034	0.0309	0.0311	0.039	0.029	0.0017	0.096	0.0987	0.190
Thymus (%)	0.331	0.365	0.345	0.327	0.332	0.0031	0.081	0.087	0.0934
Bursa (%)	0.300	0.304	0.302	0.306	0.307	0.002	0.097	0.102	0.121
**Meat composition**									
Crude protein (%)	24.79 ^b^	27.09 ^a^	26.87 ^a^	26.23 ^a^	27.19 ^a^	1.07	0.001	0.0001	0.0987
Fat (%)	7.98 ^a^	4.07 ^b^	4.67 ^b^	5.01 ^b^	5.21 ^b^	0.087	0.002	0.0001	0.0811
TAC (mmol/dL)	1.070 ^b^	1.129 ^a^	1.228 ^a^	1.197 ^a^	1.225 ^a^	0.008	0.001	0.002	0.0942

^a,b^, Means in the same row followed by different letters are significantly different at *p* ≤ 0.05. SEM, standard error of mean; TAC, total antioxidant capacity.

**Table 5:** Influence of different dietary inclusion levels of L-carnitine on microbiological intestinal contents of ducklings during 7–65 days of age (n = 5 birds/treatment).

Item	Control	L-Carnitine (mg/kg)				SEM	*p*-Value		
		50	100	200	400		Treatment	Linear	Quadratic
TBC (CFU × 10^6^)	18.03 ^a^	16.87 ^b^	15.90 ^b^	15.12 ^b^	16.65 ^b^	0.098	0.001	0.0001	0.0982
*E. coli* (CFU × 10^3^)	1.99 ^a^	1.65 ^b^	1.45 ^b^	1.59 ^b^	1.41 ^b^	0.068	0.001	0.002	0.0810
*Proteus* spp. (CFU × 10^3^)	0.997 ^a^	0.567 ^b^	0.601 ^b^	0.598 ^b^	0.700 ^b^	0.008	0.001	0.0001	0.0913
*Lactobacillus* spp. (CFU × 10^3^)	1.41 ^b^	1.90 ^a^	1.89 ^a^	1.97 ^a^	2.00 ^a^	0.002	0.002	0.001	0.109

^a,b^, Means in the same row followed by different letters are significantly different at *p* ≤ 0.05. SEM, standard error of mean; TBC, total bacterial count; *E. coli*, *Escherichia coli*; CFU, colony-forming unit.

**Table 6:** Influence of different dietary inclusion levels of L-carnitine on hematological parameters of ducklings during 7–65 days of age (n = 5 birds/treatment).

Item	Control	L-Carnitine (mg/kg)				SEM	*p*-Value		
		50	100	200	400		Treatment	Linear	Quadratic
RBC (10^6^/mm^3^)	4.05 ^b^	5.22 ^a^	5.09 ^a^	4.99 ^a^	5.01 ^a^	0.045	0.002	0.0001	0.0982
Hb (g/100 mL)	9.09 ^b^	13.89 ^a^	12.99 ^a^	12.91 ^a^	12.98 ^a^	0.078	0.001	0.002	0.078
PCV (%)	29.98 ^b^	36.08 ^a^	34.86 ^a^	35.15 ^a^	33.56 ^a^	0.385	0.001	0.001	0.102
WBC (10^3^/mm^3^)	21.89 ^b^	26.98 ^a^	27.01 ^a^	26.76 ^a^	25.89 ^a^	0.701	0.002	0.001	0.0933
Lymphocytes (%)	41.98 ^b^	48.09 ^a^	47.01 ^a^	46.89 ^a^	45.98 ^a^	0.981	0.003	0.002	0.0876
Heterophils (%)	26.87	24.98	25.12	26.09	25.89	1.09	0.091	0.110	0.092
H/L ratio	0.640	0.519	0.534	0.556	0.563	0.008	0.098	0.0829	0.0944

^a,b^, Means in the same row followed by different letters are significantly different at *p* ≤ 0.05. SEM, standard error of mean; RBC, red blood cell count; Hb, hemoglobin; PCV, packed cell volume; WBC, white blood cell count; H/L ratio, Heterophil-to-Lymphocyte ratio.

**Table 7:** Influence of different dietary inclusion levels of L-carnitine on biochemical constituents of ducklings during 7–65 days of age (n = 5 birds/treatment).

Item	Control	L-Carnitine (mg/kg)				SEM	*p*-Value		
		50	100	200	400		Treatment	Linear	Quadratic
**Protein profile (g/dL)**									
Total protein	4.45 ^b^	5.22 ^a^	5.52 ^a^	5.23 ^a^	5.43 ^a^	0.457	0.001	0.0001	0.087
Albumin	2.45	2.86	3.05	2.88	3.02	0.298	0.081	0.901	0.0978
Globulin	2.00 ^b^	2.36 ^a^	2.47 ^a^	2.35 ^a^	2.41 ^a^	0.176	0.001	0.002	0.0911
**Lipid profile (mg/dL)**									
Total lipid	488.9 ^a^	432.9 ^b^	440.9 ^b^	399.8 ^b^	411.8 ^b^	4.08	0.001	0.002	0.0981
Triglycerides	120.8 ^b^	130.8 ^a^	133.0 ^a^	132.8 ^a^	135.8 ^a^	2.28	0.001	0.0001	0.087
Cholesterol	200.0 ^a^	179.1 ^b^	181.7 ^b^	183.9 ^b^	184.1 ^b^	3.04	0.001	0.001	0.0922
HDL	30.98 ^b^	34.98 ^a^	35.92 ^a^	36.01 ^a^	35.98 ^a^	0.89	0.002	0.003	0.0871
LDL	144.9 ^a^	118.16 ^b^	119.24 ^b^	121.40 ^b^	120.95 ^b^	2.87	0.001	0.001	0.0933
**Liver function (U/L)**									
AST	66.09	64.09	65.98	67.01	66.45	1.09	0.098	0.0911	0.090
ALT	40.98	39.89	41.02	40.98	39.34	1.87	0.087	0.103	0.0866
ALP	13.09	12.99	13.11	12.87	13.98	1.09	0.097	0.0876	0.0799
**Kidney function (mg/dL)**									
Creatinine	1.09 ^a^	0.876 ^b^	0.765 ^b^	0.811 ^b^	0.812 ^b^	0.065	0.002	0.0001	0.0822
Uric acid	2.11 ^a^	1.61 ^b^	1.80 ^b^	1.72 ^b^	1.76 ^b^	0.063	0.001	0.002	0.0911
**Glucose (mg/dL) and thyroid hormones (ng/dL)**									
Glucose	160.9 ^b^	188.9 ^a^	190.6 ^a^	195.4 ^a^	193.1 ^a^	2.09	0.001	0.001	0.0911
T_3_	2.09 ^b^	2.99 ^a^	3.09 ^a^	3.11 ^a^	3.13 ^a^	0.096	0.001	0.003	0.0876
T_4_	5.78 ^b^	7.02 ^a^	7.00 ^a^	7.21 ^a^	7.09 ^a^	0.098	0.002	0.0001	0.0762

^a,b^, Means in the same row followed by different letters are significantly different at *p* ≤ 0.05. SEM, standard error of mean; HDL, high-density lipoprotein; LDL, low-density lipoprotein; AST, aspartate aminotransferase; ALT, alanine aminotransferase; ALP, Alkaline phosphatase; T_3_, triiodothyronine; T_4_, Thyroxine.

**Table 8:** Influence of different dietary inclusion levels of L-carnitine on antioxidative status and immunological indices of ducklings during 7–65 days of age (n = 5 birds/treatment).

Item	Control	L-Carnitine (mg/kg)				SEM	*p*-Value		
		50	100	200	400		Treatment	Linear	Quadratic
**Antioxidative status**									
TAC (mmol/L)	1.73 ^b^	1.90 ^a^	1.88 ^a^	1.93 ^a^	1.93 ^a^	0.098	0.001	0.001	0.0933
GSH-PX (U/L)	3.65 ^b^	3.95 ^a^	4.00 ^a^	3.90 ^a^	3.98 ^a^	0.065	0.0001	0.002	0.876
SOD (U/mL)	2.15 ^b^	2.44 ^a^	2.43 ^a^	2.36 ^a^	2.36 ^a^	0.007	0.002	0.0001	0.0790
MDA (µmol/L)	56.96 ^a^	51.92 ^b^	51.62 ^b^	50.29 ^b^	53.34 ^b^	1.65	0.001	0.003	0.0811
**Immunological indices**									
IgG (mg/dL)	388.9 ^b^	432.9 ^a^	440.9 ^a^	399.8 ^a^	411.8 ^a^	2.98	0.001	0.002	0.0922
IgM (mg/dL)	120.8 ^b^	130.8 ^a^	133.0 ^a^	132.8 ^a^	135.8 ^a^	1.79	0.001	0.001	0.0876
IgA (mg/dL)	150.0 ^b^	179.1 ^a^	181.7 ^a^	183.9 ^a^	184.1 ^a^	1.98	0.001	0.003	0.0934
PA (%)	30.98 ^b^	34.98 ^a^	35.92 ^a^	36.01 ^a^	35.98 ^a^	1.098	0.002	0.001	0.0890
PI (%)	144.9 ^a^	118.16 ^b^	119.24 ^b^	121.40 ^b^	120.95 ^b^	1.67	0.001	0.001	0.0902
BA (%)	20.87 ^b^	22.78 ^a^	23.90 ^a^	22.98 ^a^	23.09 ^a^	0.87	0.001	0.002	0.102
LA (%)	7.21 ^b^	8.34 ^a^	9.00 ^a^	8.98 ^a^	9.04 ^a^	0.06	0.002	0.003	0.0922
LTT (%)	17.01 ^b^	19.98 ^a^	21.09 ^a^	20.99 ^a^	22.02 ^a^	0.98	0.001	0.0001	0.065

^a,b^, Means in the same row followed by different letters are significantly different at *p* ≤ 0.05. SEM, standard error of mean; TAC, total antioxidant capacity; GSH-Px, glutathione peroxidase; SOD, superoxide dismutase; MDA, malondialdehyde; IgG, immunoglobulin G; IgM, immunoglobulin M; IgA, immunoglobulin A; PA, phagocytic activity; PI, phagocytic index; BA, bactericidal activity; LA, lysozyme activity; LTT, lymphocyte transformation test.

## Data Availability

The supplementary data can be made available from the first corresponding author upon reasonable request.
